# Social contact and inequalities in depressive symptoms and loneliness among older adults: A mediation analysis of the English Longitudinal Study of Ageing

**DOI:** 10.1016/j.ssmph.2021.100726

**Published:** 2021-01-12

**Authors:** Michael J. Green, Elise Whitley, Claire L. Niedzwiedz, Richard J. Shaw, S. Vittal Katikireddi

**Affiliations:** a MRC/CSO Social & Public Health Sciences Unit, University of Glasgow, UK; b Institute of Health & Wellbeing, University of Glasgow, UK; c Public Health Scotland, UK

**Keywords:** Social contact, Depressive symptoms, Loneliness, Older adults

## Abstract

**Background:**

Social contact, including remote contact (by telephone, email, letter or text), could help reduce social inequalities in depressive symptoms and loneliness among older adults.

**Methods:**

Data were from the 8th wave of the English Longitudinal Study of Aging (2016/17), stratified by age (n = 1578 aged <65; n = 4026 aged 65+). Inverse probability weighting was used to estimate average effects of weekly in-person and remote social contact on depressive symptoms (score of 3+ on 8-item CES-D scale) and two measures of loneliness (sometimes/often feels lonely vs hardly ever/never; and top quintile of UCLA loneliness scale vs all others). We also estimated controlled direct effects of education, partner status, and wealth on loneliness and depressive symptoms under two scenarios: 1) universal infrequent (<weekly) in-person social contact; and 2) universal weekly remote social contact.

**Results:**

Weekly in-person social contact was associated on average with reduced odds of loneliness, but associations with remote social contact were weak. Lower education raised odds of depressive symptoms and loneliness, but differences were attenuated with infrequent in-person contact. Respondents living alone experienced more depressive symptoms and loneliness than those living with a partner, and less wealth was associated with more depressive symptoms. With universal infrequent in-person contact, these differences narrowed among those aged under 65 but widened among those aged 65+. Universal weekly remote contact had relatively little impact on inequalities.

**Conclusions:**

Reduced in-person social contact may increase depressive symptoms and loneliness among older adults, especially for those aged 65+ who live alone. Reliance on remote social contact seems unlikely to compensate for social inequalities.

## Highlights

• In-person but not remote contact associated with less depression/loneliness.
• In-person contact < weekly reduces educational inequalities in depression/loneliness.
• In those aged 65+ partner/wealth inequalities widen with <weekly in-person contact.
• Weekly remote contact has little impact on inequalities in depression/loneliness.

## 1 Introduction

### 1.1 Background

Many older adults experience depressive symptoms and loneliness (Age UK, 2011; Green & Benzeval, 2011; Hansen & Slagsvold, 2016; Meeks et al., 2011; Niedzwiedz et al., 2016), which are correlated but distinct phenomena that may share some common causes (Heinrich & Gullone, 2006). Both are associated with lower quality of life, health-risk behaviours and poor physical health (Beutel et al., 2017; Courtin & Knapp, 2017; Eaton et al., 2008; Meeks et al., 2011; Rodda et al., 2011; Shankar et al., 2011; Valtorta et al., 2016), and are socially patterned, being more common among older adults from poorer socioeconomic backgrounds and who live alone (Age UK, 2011; Beutel et al., 2017; Chang-Quan et al., 2010; Green & Benzeval, 2011; Hansen & Slagsvold, 2016; Kamiya et al., 2013; Meeks et al., 2011; Niedzwiedz et al., 2016). Social mitigation responses to the worldwide COVID-19 pandemic, emphasising physical distance from others, may exacerbate these issues (Armitage & Nellums, 2020; Douglas et al., 2020; Holmes et al., 2020).

Social contact with friends and family may contribute to inequalities in loneliness and depressive symptoms. Social contact may be in-person or remote (i.e. via phone/internet etc.), but is distinct from loneliness, which is a perceived feeling of social isolation (Age UK, 2011; Courtin & Knapp, 2017; Heinrich & Gullone, 2006; Hughes et al., 2004; Niedzwiedz et al., 2016). One can feel lonely amid frequent social contacts, or not feel lonely with very few social contacts. Nevertheless, more frequent social contact is associated with less loneliness and fewer depressive symptoms (Age UK, 2011; Kearns et al., 2015; Niedzwiedz et al., 2016; Teo et al., 2015), and is more common among older people who are socioeconomically advantaged or who live with a partner (Ajrouch et al., 2005; Gray, 2009; Kearns et al., 2015).

The nature of social contact continues to change in response to technological developments in digital communications but impacts of such advancements will not necessarily be felt equally. Remote contact has become especially salient during the COVID-19 pandemic. Many countries have enacted ‘lockdown’ social mitigation measures, where reduced physical proximity to others is intended to slow the infection transmission rate, but such measures are likely to have psychological impacts (Armitage & Nellums, 2020; Brooks et al., 2020; Douglas et al., 2020; Sood, 2020). Identified as high risk for COVID-19, older adults have been advised to follow stringent social distancing measures to avoid infection (Armitage & Nellums, 2020; Douglas et al., 2020). Maintaining or increasing remote social contact has been promoted to mitigate impacts of social distancing (Armitage & Nellums, 2020; Brooks et al., 2020; Chatterjee & Yatnatti, 2020; Sood, 2020), but remote contact has been less strongly associated with depressive symptoms and loneliness than in-person contact (Teo et al., 2015), so may not compensate adequately. We theorise that socioeconomically disadvantaged compared to advantaged adults may differ not only in the prevalence of in-person and remote social contacts, but also in their susceptibility to the effects of in-person and remote social contacts (Diderichsen et al., 2019). Changes in social contact could therefore potentially either narrow inequalities (by reducing social patterning of salutary factors) or widen them (if some benefit more from contact than others) (Niedzwiedz et al., 2016). Similar arguments could be made for those who live alone compared to those who live with a partner. Understanding the different contributions that in-person and remote social contacts make to inequalities in loneliness and depressive symptoms is important in informing policy responses to improve health, both with regards to mitigation of social distancing, and with digital communications being increasingly important.

### 1.2 Research questions

We use data from the English Longitudinal Study of Aging, to estimate answers to the following questions:

1. What are the effects of in-person and remote social contact on loneliness and depressive symptoms?
2. To what extent are inequalities in loneliness and depressive symptoms affected by making in-person social contact infrequent for everyone?
3. To what extent are inequalities in loneliness and depressive symptoms affected by making remote social contact frequent for everyone?

## 2 Methods

### 2.1 Sample

**Fig. 1:**
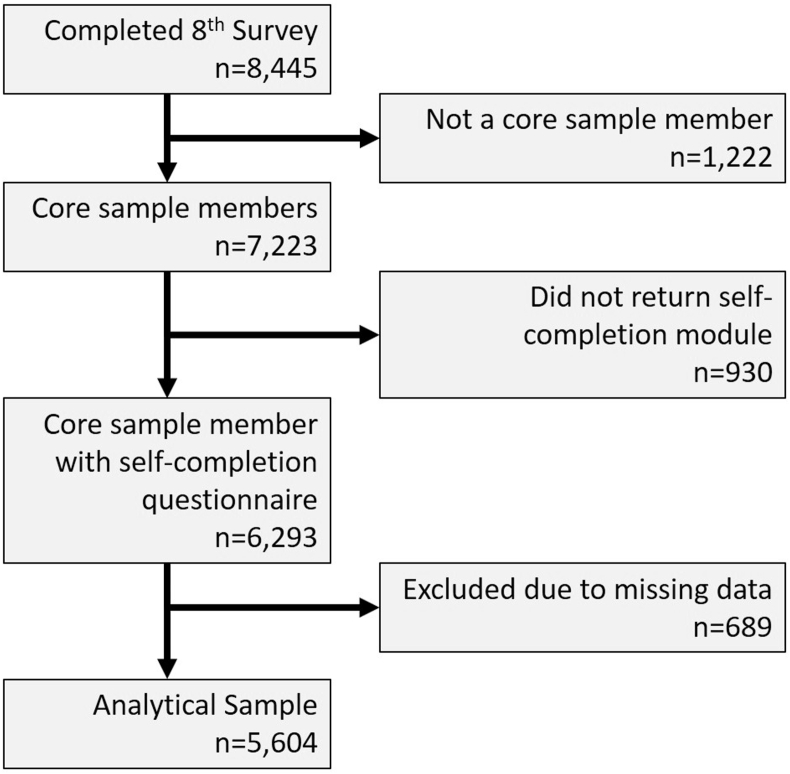
Derivation of analytical sample.

The English Longitudinal Study of Ageing (ELSA) is a large-scale, representative, longitudinal panel study of people aged 50 and over living in private households in England (Marmot et al., 2018). The core sample has been drawn from respondents to the Health Survey for England (HSE) since 1998. We focus on 7223 core sample members who were interviewed at the most recent survey wave (wave 8: 2016/2017; 82.4% of those who were eligible for inclusion because they were still alive and living in the UK). We excluded 930 respondents who did not return a self-completion questionnaire, and a further 535 respondents with missing data on relevant variables at wave 8 and 154 respondents who lacked data on relevant measures from the previous wave (see Fig. 1). This resulted in a final sample of 5604 respondents (77.6% of the core sample members interviewed).

### 2.2 Measures

#### 2.2.1 Loneliness and depressive symptoms

Depressive symptoms were identified with the 8-item version of the Centre for Epidemiologic Studies Depression Scale (CES-D; Radloff, 1977), with scores ranging from 0 (least depressed) to 8 (most depressed). Scores of 3+ were coded as indicating high levels of depressive symptoms. Loneliness at wave 8 was assessed in two ways. Firstly, we considered responses to the self-complete questionnaire question “How often do you feel lonely?” (some of the time/often versus hardly ever/never). Secondly we used the 3-item UCLA loneliness scale (Hughes et al., 2004), which incorporates responses to three questions: “How often do you feel that you lack companionship?”; “How often do you feel left out?”; and “How often do you feel isolated from others?”. Scores ranged between 3 and 9 and we compared the loneliest quintile (scores 6–9) versus the other four (scores 3–5). Analyses using continuous scores are including as supplementary information.

#### 2.2.2 Primary exposures

We considered three potential sources of inequality in loneliness and depressive symptoms: 1) education, compared respondents with A level equivalent or higher qualifications (i.e. beyond completion of ordinary schooling) versus those with lesser or no qualifications; 2) partner status, comparing those living with a partner (regardless of marital status) versus those not living with a partner; and 3) wealth, measured using quintiles of net total non-pension household wealth (a summary of the total value of the financial, physical and housing wealth owned by the household; derivation of this variable has been described in full elsewhere) (Demakakos et al., 2016). For wealth, we focus on results comparing the least to the wealthiest quintile (with results for other quintiles included as supplementary information).

#### 2.2.3 Social contact

Respondents were asked how often, on average, they met up with their children, other family members or friends (separate questions for each), and we compared those meeting up with at least one of their children, other family members or friends at least weekly versus those meeting up less frequently or reporting no children, family or friends. Weekly remote contact was coded similarly using questions on frequency of contact by telephone, letter, email or text with children, other family or friends (again separate questions were asked for each).

#### 2.2.4 Confounding variables

Confounding variables included: sex, five-year age group, ethnicity (white versus ethnic minority), government office region, any children in household (yes/no), any children outside household (yes/no), number of people in household other than respondent and partner, whether respondent felt close to their children, other family or friends, housing tenure (owner-occupant/rented), economic activity (in paid employment/not), social class (I/II, III non-manual, III manual, IV/V) of last known occupation coded according to the British Registrar General's scheme which indicates occupational differences in status and economic resources (Galobardes et al., 2006; Office of Population Censuses and Surveys, 1980), number of problems with instrumental activities of daily living, indicating physical and mental functional capacity (Lawton & Brody, 1969), and whether the respondent was a member of any clubs or organisations. We also included identical measures of social contact, depressive symptoms and loneliness from the previous wave of the survey (2 years earlier).

### 2.3 Statistical analyses

All models used weights to adjust for sampling and drop-out (Marmot et al., 2018). In preliminary analyses we checked whether associations between the exposures and the outcome differed considerably by age and gender. This indicated an interaction with age, so we stratified our analyses between those aged under 65 (the UK retirement age) and those aged 65 or more.

For research question 1 we separately estimated the effects of in-person and remote social contact on loneliness and depressive symptoms, using inverse probability weighting (IPW) (Austin, 2011). This is described more fully in the Appendix (section 5.1) but adjusts for measured potential confounders (including education, partner status and wealth, as well as the other confounders listed in section 2.2.4). Estimates represent average effects of in-person and remote social contact on depressive symptoms and loneliness within the sample (assuming no residual confounding or reverse causation).

**Fig. 2:**
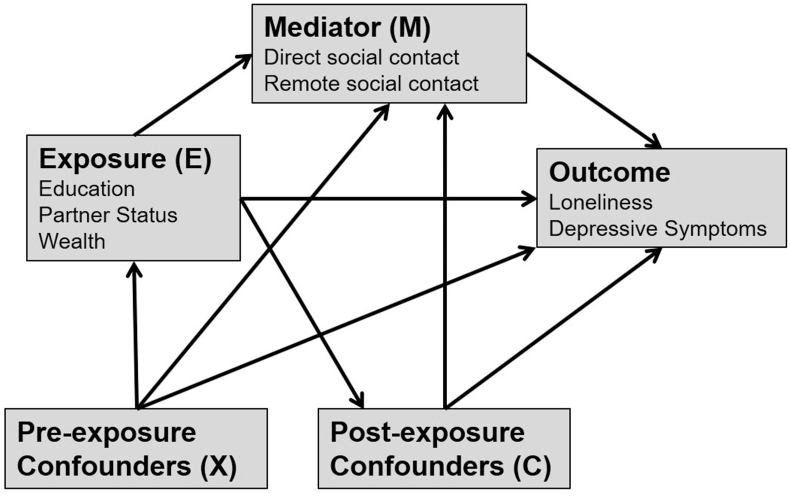
Assumptions about causal direction in our analyses.

For research questions 2 and 3, we assume social contact may mediate effects of either education, partner status or wealth on loneliness and depressive symptoms outcomes, as shown in Fig. 2. We distinguish pre- and post-exposure confounders. Pre-exposure confounders (X) are potential common causes of the exposure, the mediator and the outcome. In contrast, post-exposure confounders (C) are potential common causes of the mediator and the outcome but may (or may not) be caused by the exposure. This distinction is important for estimating the effect of the exposure after intervention on the mediator (VanderWeele, 2009). Table 1 shows which variables were considered as pre/post confounders depending on the exposure in question. Given ambiguity regarding causal direction between partner status and socioeconomic variables (housing tenure, social class, economic activity and wealth) we performed sensitivity analyses with these variables re-positioned as pre-exposure confounders.

**Table 1:** Analysis variables.

Exposure (E)	Pre-Exposure Confounders (X)	Post-Exposure Confounders (C)
Education	Gender	Remote/In-person social contact^a^
	Age	Partner status
	Ethnicity	Wealth
	Region	Child in Household
		Child out of Household
		Other Adults in Household
		Close to Children
		Close to Family
		Close to Friends
		Housing Tenure
		Economic Activity
		Social Class
		IADL
		Member of social organisation
		Remote/In-person social contact (previous wave)
		Loneliness (previous wave)
		Depressive Symptoms (previous wave)
Partner status	Gender	Remote/In-person social contact^a^
	Age	Wealth^b^
	Ethnicity	Child in Household
	Region	Child out of Household
	Education	Other Adults in Household
		Close to Children
		Close to Family
		Close to Friends
		Housing Tenure^b^
		Economic Activity^b^
		Social Class^b^
		IADL
		Member of social organisation
		Remote/In-person social contact (previous wave)
		Loneliness (previous wave)
		Depressive Symptoms (previous wave)
Wealth	Gender	Remote/In-person social contact^a^
	Age	Child in Household
	Ethnicity	Child out of Household
	Education	Other Adults in Household
	Partner status	Close to Children
	Housing Tenure	Close to Family
	Economic Activity	Close to Friends
	Social Class	IADL
		Member of a social organisation
		Remote/In-person social contact (previous wave)
		Loneliness (previous wave)
		Depressive Symptoms (previous wave)

IADL=Instrumental Activities of Daily Living.

a Depending on whether in-person or remote social contact was the mediator of interest, the other was included as a post-exposure confounder.

b Since the causal direction between partner status and these socioeconomic variables was particularly ambiguous we performed sensitivity analyses where these variables were re-positioned as pre-exposure confounders.

Total exposure effects were estimated using IPW (see Appendix 5.2) to estimate the average effects of education, partner status or wealth on depressive symptoms and loneliness, adjusting for pre-exposure confounders. These were compared to estimates of controlled direct effects (CDEs) for each exposure on loneliness and depressive symptoms, which were estimated using inverse-probability weighted marginal structural models to adjust appropriately for pre- and post-exposure confounders (see Appendix 5.3; VanderWeele, 2009). Biases related to exposure-mediator interaction and mediator-outcome confounders that are affected by the exposure are known issues with more traditional mediation methods (Richiardi et al., 2013; VanderWeele, 2009). The CDE represents the estimated effect of the exposure under a hypothetical intervention to set the mediator (social contact) to the same value for all respondents. Estimation allows for an interaction between the exposure and social contact (e.g. if social contact is more beneficial for some groups than others), such that CDEs can differ depending on the level that social contact is set to. We estimated CDEs for two scenarios: 1) universal infrequent (<weekly) in-person social contact; and 2) universal weekly remote social contact. We do not specifically attempt to estimate indirect effects via social contact because this requires more stringent assumptions and/or complex methodology while the CDE represents what we are most interested in (Naimi et al., 2014; VanderWeele, 2009), i.e. how inequalities may be impacted by interventions affecting social contact.

## 3 Results

### 3.1 Sample characteristics

Supplementary Table 1 presents characteristics of the analytical sample by age. Respondents aged 65 or more (n = 4026) were more likely to be white, living alone, less educated, and have frequent in-person social contact than those aged under 65 (n = 1578). Supplementary Table 1 also summarises characteristics of those who were excluded due to missing data, who were more likely to be at the extremes of the age range, from ethnic minority groups, living with a partner, less educated, less wealthy, have less frequent in-person and remote social contact, and were more likely to feel lonely or depressed.

### 3.2 Frequency of social contact

Table 2 shows observed proportions within each age group and exposure category experiencing less than weekly in-person and remote social contact. Infrequent in-person social contact was more common in both age groups than infrequent remote social contact. Infrequent in-person social contact was most common among those with more education, who lived with a partner, or were wealthier. These differences were more pronounced in the 65+ age group, and the wealth difference was in the opposite direction for the under 65 age group. There was little social patterning of remote social contact by education, and the direction of patterning by partner status differed by age. Infrequent remote contact was more common in the lowest than the highest wealth quintile in the under 65 age group.

**Table 2:** Proportions experiencing infrequent in-person and remote social contact by education, partner status and wealth.

	<Weekly in-person social contact (%)		<Weekly remote social contact (%)	
	Age <65	Age 65+	Age <65	Age 65+
**Education**				
Low	26.6	22.2	11.3	10.4
High	28.9	27.6	11.8	11.8
**Partner status**				
Lives alone	27.2	17.5	15.4	8.7
With partner	27.8	27.7	10.2	12.1
**Wealth**				
Lowest Quintile	30.2	20.2	17.2	11.0
Highest Quintile	25.7	29.4	7.7	10.1
**Total**	27.6	24.3	11.5	11.0

### 3.3 Estimated effects of social contact

Table 3 shows estimates of the average effects of in-person and remote social contact on loneliness and depressive symptoms. Depressive symptoms were not associated with weekly in-person contact in either age group, but weekly in-person social contact was associated with reduced odds of loneliness in both age groups, even after adjustment for confounders (albeit not consistently across both loneliness measures). There were no clear associations with remote social contact after adjustment for confounders. Findings using continuous scores for depressive symptoms and loneliness were similar (Supplementary Table 2).

**Table 3:** Estimates of effects of in-person and remote social contact on depressive symptoms and loneliness.

	Weekly in-person social contact (vs. less than weekly)				Weekly remote social contact (vs. less than weekly)			
	Age <65 *N = 1578*		Age 65+ *N = 4026*		Age <65 *N = 1578*		Age 65+ *N = 4026*	
	OR	95% CI	OR	95% CI	OR	95% CI	OR	95% CI
*CES-D Depressive symptoms*								
Sample weighted association	0.76	0.52–1.10	1.02	0.83–1.25	0.60	0.37–0.96	1.13	0.84–1.54
ATE estimate^a^	0.88	0.51–1.51	1.00	0.73–1.35	0.76	0.33–1.79	0.93	0.54–1.62
*Sometimes/Often Feels Lonely*								
Sample weighted association	0.71	0.52–0.98	0.89	0.75–1.06	0.83	0.53–1.29	0.84	0.66–1.08
ATE estimate^a^	0.64	0.41–1.00	0.84	0.65–1.10	0.65	0.28–1.47	0.85	0.55–1.33
*UCLA Loneliness*								
Sample weighted association	0.57	0.40–0.81	0.68	0.56–0.83	0.54	0.34–0.86	0.65	0.50–0.85
ATE estimate^a^	0.71	0.43–1.16	0.70	0.52–0.94	0.55	0.22–1.43	0.68	0.41–1.12

a ATE: Average treatment effect, i.e. the estimated average effect of weekly social contact within the sample after adjusting for education, partner status, wealth and all other confounders listed in Table 1. These estimates assume no residual confounding or reverse causation.

### 3.4 Estimated effects of education, partner status and wealth

Table 4 shows ATE and CDE estimates of education, partner status and wealth on depressive symptoms and loneliness for those both below and over the age of 65. Findings were largely similar with continuous measures of depressive symptoms and loneliness (Supplementary Table 3). Lower education was associated with more loneliness and depressive symptoms in both age groups, and the CDE estimates suggested that universally infrequent in-person social contact would attenuate this inequality. CDE estimates of ensuring weekly remote contact for everyone also indicated attenuation of this inequality but clear differences remained for sometimes/often feeling lonely, and for depressive symptoms in those aged 65+.

**Table 4:** Estimates for effects of education, partner status and wealth on depressive symptoms and loneliness.

	Low Education (vs High Education)				Lives Alone (vs with Partner)				Lowest Wealth Quintile (vs Highest Quintile)			
	Age <65 *N = 1578*		Age 65+ *N = 4026*		Age <65 *N = 1578*		Age 65+ *N = 4026*		Age <65 *N = 1578*		Age 65+ *N = 4026*	
	OR	95% CI	OR	95% CI	OR	95% CI	OR	95% CI	OR	95% CI	OR	95% CI
*CES-D Depressive symptoms*												
ATE estimate^a^	1.59	1.11–2.29	1.50	1.24–1.81	4.02	2.81–5.75	2.02	1.66–2.46	6.82	2.41–19.28	2.90	1.12–7.53
CDE estimate^b^ (<weekly in-person contact)	1.57	0.64–3.86	1.00	0.56–1.79	2.58	0.97–6.86	1.72	0.93–3.17	2.04	0.22–18.99	4.28	1.22–15.02
CDE estimate^b^ (weekly remote contact)	0.87	0.46–1.65	1.35	1.06–1.73	6.21	3.08–12.49	1.91	1.42–2.56	5.19	1.52–17.70	2.35	0.87–6.33
*Sometimes/Often Feels Lonely*												
ATE estimate^a^	1.44	1.07–1.93	1.44	1.23–1.69	4.94	3.57–6.82	4.41	3.71–5.26	2.73	0.79–9.49	0.47	0.12–1.78
CDE estimate^b^ (<weekly in-person contact)	1.02	0.48–2.20	1.30	0.79–2.14	3.56	1.56–8.14	5.37	2.99–9.63	2.35	0.41–13.27	0.91	0.26–3.25
CDE estimate^b^ (weekly remote contact)	1.24	1.12–7.43	1.33	1.07–1.66	6.30	3.63–10.93	4.66	3.62–5.98	1.62	0.39–6.70	0.38	0.10–1.48
*UCLA Loneliness*												
ATE estimate^a^	1.48	1.04–2.10	1.35	1.12–1.62	4.91	3.42–7.06	3.55	2.92–4.32	2.93	0.71–12.12	2.50	1.01–6.20
CDE estimate^b^ (<weekly in-person contact)	1.27	0.58–2.75	1.09	0.62–1.90	2.61	1.13–6.03	6.06	3.35–10.96	0.60	0.11–3.38	1.05	0.32–3.45
CDE estimate^b^ (weekly remote contact)	1.06	0.55–2.02	1.62	0.90–1.50	6.47	3.28–12.75	3.74	2.82–4.96	1.76	0.36–8.69	2.08	0.80–5.46

a ATE: Average Treatment Effect, i.e. the estimated average effect of each exposure, after adjusted for pre-exposure confounders (see Table 1). These estimates (and the CDE estimates) assume no residual confounding or reverse causation.

b CDE: Controlled Direct Effect, i.e. an estimate of the effect of each exposure if social contact were set to the value shown in parentheses. CDE estimates allow for interactions between social contact and the exposure and additionally adjust for differences in post-exposure confounders that were not due to the exposure (see Table 1).

Living alone was associated with more depressive symptoms and loneliness than living with a partner. CDE estimates for universal infrequent in-person social contact indicated that differences in loneliness would widen among those aged over 65 but narrow among those aged under 65, while inequalities in depressive symptoms were attenuated in both age groups. CDE estimates for ensuring weekly remote contact for everyone indicated relatively little impact on these inequalities in those aged 65+ and there was some indication of inequalities widening among those aged under 65. Findings for partner status were similar with socioeconomic confounders re-positioned as pre-exposure confounders (Supplementary Table 4).

Being in the least wealthy compared to the wealthiest quintile was not clearly associated with loneliness except for the UCLA loneliness measure among the 65+ age group but was associated with greater odds of depressive symptoms in both age groups. CDE estimates for universal infrequent in-person social contact indicated attenuation of this inequality in depressive symptoms among those aged under 65 and the inequality in loneliness among those aged 65+, but increased inequalities in depressive symptoms among those aged 65+. CDE estimates of ensuring weekly remote contact for everyone indicated some attenuation of the inequalities in loneliness and depressive symptoms among the 65+ age group, but little impact on the inequality in depressive symptoms among those aged under 65. Results for other wealth quintiles (Supplementary Table 5) were similar, though there was not a clear linear wealth gradient.

## 4 Discussion

### 4.1 Summary of findings

On average, in-person social contact appeared associated with some reduced risk of loneliness in older adults in England, while estimated effects of remote social contact were relatively weak. Nevertheless, estimating effects of restricting everyone to infrequent (<weekly) in-person social contact suggested narrower inequalities in depressive symptoms and loneliness by education. Inequalities in depressive symptoms and loneliness by partner status and in depressive symptoms by wealth were impacted differently depending on age, with restricted in-person contact narrowing these inequalities in those aged <65 but widening them among adults aged 65+. Estimates for ensuring everyone received weekly remote contact also indicated some attenuation of inequalities but impacts were relatively weak compared to restricted in-person contact.

### 4.2 Implications

Our findings are consistent with others showing associations between social contact and depressive symptoms and loneliness (Age UK, 2011; Kearns et al., 2015; Niedzwiedz et al., 2016), with in-person contact more important than remote (Teo et al., 2015). While others found social contacts concentrated among the more advantaged (Ajrouch et al., 2005; Gray, 2009; Kearns et al., 2015), we found weekly in-person contact was less common among more advantaged adults, especially those aged 65+. Considering this and that in-person social contact was on average associated with less loneliness, the narrowing of inequalities indicated by our CDE estimates (which allow for interaction between social contact and education) suggests heterogeneity in the impact of in-person social contact, with those of higher education deriving greater benefit. While we did adjust for close relationships with friends and other family members, this could be due to differences in the quality of contacts, or differences in frequency of contact beyond the weekly threshold used.

Our findings suggest that reductions in in-person social contact, e.g. under social mitigation measures related to COVID-19, are likely to lead on average to increases in loneliness among older adults. However, impacts may be experienced unequally and we estimated that those aged 65+ who live alone were particularly at risk for depressive symptoms and loneliness under restrictions to in-person contact. This has been anticipated (Armitage & Nellums, 2020; Douglas et al., 2020), with remote social contact advised to mitigate these impacts (Brooks et al., 2020; Chatterjee & Yatnatti, 2020; Sood, 2020). We found that remote contact, at least as experienced pre-COVID-19, seemed unlikely to compensate, with relatively little effect on depressive symptoms or loneliness, or on inequalities in depressive symptoms and loneliness by education, partner status or wealth (even when assuming no disparities in establishing regular remote contact).

### 4.3 Limitations

Depressive symptoms and loneliness were modelled here as separate outcomes. The two are known to be related (Heinrich & Gullone, 2006) and the CES-D scale used to measure depressive symptoms even includes an item on feeling lonely (Radloff, 1977). Thus, where similar associations were observed, this could be consistent with both common causation, or with effects on one via the other. Furthermore, our partner status measure compared living with a partner to not living with a partner, with the latter potentially including those with no partner and those not living with their partner. Relationships with a non-resident romantic partner could potentially have been captured as in-person or remote social contact, depending on how respondents interpreted the term “friend”.

While adjustment was made for earlier measures of social contact depression and loneliness, this was as post-exposure confounders, i.e. assuming that education, partner status and wealth were causally precedent), and we assumed a causal direction from social contact to depressive symptoms and loneliness where these were measured concurrently. Our results could also be accounted for by depressive symptoms or loneliness affecting concurrent measures of social contact, or by residual confounding.

Our estimates based on pre-COVID-19 data may not necessarily generalise. For example, infrequent social contact under COVID-19 mitigation measures, may have different effects to infrequent social contact experienced under other circumstances (Fancourt & Steptoe, 2020). Moreover, the COVID-19 situation may have prompted qualitative improvements in remote contact that alter its effect. Our results, which estimate effects of remote contact as it was experienced pre-COVID-19, should not be taken to mean that efforts to provide quality remote social contact (e.g. see Van Orden et al., 2020; Zubatsky et al., 2020) are likely to be ineffective, but highlight the importance of better understanding how benefit can be derived from remote contact, and how effective forms of remote contact might impact on inequalities. While the COVID-19 pandemic enhances the salience of these issues, continuing increases in use of digital communications mean they will remain important after the pandemic.

## 4.4 Conclusions

Reductions in in-person social contact could on average result in increased loneliness among older adults. Adults aged 65+ who lived alone appeared especially vulnerable to reductions in social contact, with greater estimated increases in risk for depressive symptoms and loneliness than similarly aged adults living with a partner. Inequalities in depressive symptoms and loneliness by education could narrow as in-person contact is reduced, as those with more education seemed to derive more benefit from contact. Remote social contact seemed insufficient to mitigate adverse impacts or inequalities, and more attention is needed into how remote contact can become more efficacious, or to other strategies for addressing inequalities in depressive symptoms and loneliness among older adults.

## 5 Appendix

### 5.1 Estimating effects of social contact

Average Treatment Effects (ATEs) were estimated by regressing outcomes on each social contact variable, with each respondent assigned a weight equal to P(M)/P(M|E,X,C). M is their observed level of social contact. E represents the main exposure variables (education, partner status and wealth), which are confounders for the effect of social contact. X and C represent sets of pre and post-exposure confounders as listed in Table 1 (the pre/post distinction is not important here but is important for estimating impacts on inequalities below). The purpose of this weighting is to balance observed confounders (E, X and C) across levels of M. The probabilities required to calculate the weights were estimated via logistic regression models of M (with and without E, X and C).

### 5.2 Estimating total effects of exposure

Outcomes were regressed on each exposure (education, partner status, or wealth) with each respondent assigned a weight as P(E)/P(E|X), where E represents respondents’ observed values for the exposure in question, and X represents pre-exposure confounders as listed in Table 1. The purpose of this weighting is to balance the confounders (X) across levels of E. The probabilities required to calculate the weights were estimated via logistic regression models of E (with and without X).

### 5.3 Estimating controlled direct effects

Outcomes were regressed on the exposure (education, partner status or wealth), mediator (in-person or remote social contact), and an interaction term for the two factors. These marginal structural models were weighted using a combination of the weight for the total effect of the exposure (as described above; P(E)/P(E|X)) and a modified version of the social contact weight, this time calculated as P(M|E)/P(M|E,X,C). As the numerator of this weight is conditional on the exposure in question (E), it serves to balance intermediate confounders (C), but only within levels of the exposure, so differences in C that are due to the exposure are retained. The probabilities required to calculate these weights were estimated via logistic regression models of E and M (conditioned on E, X and/or C as required). This means estimates are adjusted for differences in pre-exposure confounders, and for differences in post-exposure confounders that are not due to the exposure. In contrast with traditional regression analyses, differences in post-exposure confounders that *are* due to the exposure are not adjusted out, and the indirect effect of the exposure on the outcome via C in Fig. 2 is included.

## Ethical statement

The paper is based on secondary analysis of data that was subject to ethical review at the point of collection.

## CRediT authorship contribution statement

**Michael J. Green:** Conceptualization, Methodology, Visualisation, Writing - original draft, Writing - review & editing. **Elise Whitley:** Conceptualization, Methodology, Data curation, Investigation, Formal analysis, Writing - original draft, Writing - review & editing. **Claire L. Niedzwiedz:** Methodology, Writing - review & editing. **Richard J. Shaw:** Methodology, Writing - review & editing. **S. Vittal Katikireddi:** Conceptualization, Methodology, Writing - review & editing.

## Declaration of competing interest

None.

## Footnotes

Appendix A Supplementary data to this article can be found online at https://doi.org/10.1016/j.ssmph.2021.100726.
