# Supplementary material for: Social contact and inequalities in depressive symptoms and loneliness among older adults: A mediation analysis of the English Longitudinal Study of Ageing
Source: SSM Popul Health. 2021 Jan 12;13:100726. doi: 10.1016/j.ssmph.2021.100726 (PMC7820553; doi:10.1016/j.ssmph.2021.100726)
Supplement: Multimedia component 1 [file mmc1.docx]

**Supplementary Table 1: Characteristics of analytical sample by age group**

|  | **Analytical sample:**  **Age <65**  **(N=1,578)** | **Analytical sample:**  **Age 65+**  **(N=4,026)** | **Analytical sample:**  **All Ages**  **(N=5,604)** | **Excluded for missing data^a^**  **(N=689)** |
| --- | --- | --- | --- | --- |
|  |  |  |  |  |
| **Sex (%)** |  |  |  |  |
| Female  Male | 56.9  43.1 | 55.6  44.4 | 56.0  44.0 | 57.7  42.3 |
|  |  |  |  |  |
| **Age (%)** |  |  |  |  |
| <60  60-64  65-69  70-74  75-79  80+ | 34.6  65.4  -  -  -  - | -  -  31.9  26.3  20.1  21.7 | 9.7  18.4  23.0  18.9  14.5  15.6 | 20.3  16.4  17.3  16.7  10.2  19.4 |
|  |  |  |  |  |
| **Ethnicity (%)** |  |  |  |  |
| White  Ethnic Minority | 95.8  4.2 | 98.0  2.0 | 97.4  2.6 | 95.9  4.1 |
|  |  |  |  |  |
| **Lives with partner (%)** |  |  |  |  |
| Yes  No | 74.1  25.9 | 66.6  33.4 | 68.8  31.3 | 70.2  29.8 |
|  |  |  |  |  |
| **Highest educational qualification (%)** |  |  |  |  |
| A level equivalent or higher  O level equivalent or lower | 46.1  53.9 | 39.5  60.5 | 41.4  58.6 | 38.0  62.0 |
|  |  |  |  |  |
| **Wealth quintiles (%)** |  |  |  |  |
| 1 (lowest)  2  3  4  5 (Highest) | 20.6  19.1  18.2  20.7  21.5 | 17.4  20.1  21.2  21.1  20.2 | 18.3  19.8  20.3  21.0  20.6 | 22.9  22.5  22.4  18.2  14.1 |
|  |  |  |  |  |
| **At least weekly in-person contact (%)** |  |  |  |  |
| No  Yes | 27.6  72.4 | 24.3  75.7 | 25.3  74.8 | 27.1  72.9 |
|  |  |  |  |  |
| **At least weekly remote contact (%)** |  |  |  |  |
| No  Yes | 11.5  88.5 | 11.0  89.0 | 11.1  88.9 | 12.2  87.8 |
|  |  |  |  |  |
| **How often feels lonely (%)** |  |  |  |  |
| Hardly ever/never  Some of time/often | 70.2  29.8 | 71.5  28.5 | 71.1  28.9 | 64.4  35.6 |
|  |  |  |  |  |
| **CES-D (%)** |  |  |  |  |
| <3  3+ | 81.9  18.1 | 81.5  18.5 | 81.6  18.4 | 79.6  20.4 |
|  |  |  |  |  |
| **UCLA Loneliness score** |  |  |  |  |
| Bottom 4 quintiles  Top quintile | 81.1  18.9 | 81.3  18.7 | 81.3  18.7 | 78.3  21.7 |
|  |  |  |  |  |

^a^Figures in this column are based on those who had valid data for the characteristic in question.
