# Supplementary material for: Social contact and inequalities in depressive symptoms and loneliness among older adults: A mediation analysis of the English Longitudinal Study of Ageing
Source: SSM Popul Health. 2021 Jan 12;13:100726. doi: 10.1016/j.ssmph.2021.100726 (PMC7820553; doi:10.1016/j.ssmph.2021.100726)
Supplement: Multimedia component 2 [file mmc2.docx]

**Supplementary Table 2: Continuous score estimates of effects of in-person and remote social contact on depressive symptoms and loneliness**

|  | **Weekly in-person social contact**  **(vs. less than weekly)** | | | | **Weekly remote social contact**  **(vs. less than weekly)** | | | |
| --- | --- | --- | --- | --- | --- | --- | --- | --- |
|  | *Age <65*  *N=1,635* | | *Age 65+*  *N=4,123* | | *Age <65*  *N=1,635* | | *Age 65+*  *N=4,123* | |
|  | ß | 95% CI | ß | 95% CI | ß | 95% CI | ß | 95% CI |
| *CES-D Depressive symptoms* |  |  |  |  |  |  |  |  |
| Sample weighted association | -0.27 | -0.59, 0.06 | 0.01 | -0.13, 0.16 | -0.62 | -1.10, -0.13 | -0.00 | -0.24, 0.23 |
| ATE estimate^a^ | -0.15 | -0.58, 0.27 | -0.05 | -0.27, 0.18 | -0.31 | -0.95, 0.33 | -0.09 | -0.57, 0.39 |
| *UCLA Loneliness* |  |  |  |  |  |  |  |  |
| Sample weighted association | -0.45 | -0.70, -0.19 | -0.27 | -0.39, -0.14 | -0.51 | -0.89, -0.13 | -0.34 | -0.53, -0.16 |
| ATE estimate^a^ | -0.25 | -0.58, 0.08 | -0.26 | -0.44, -0.07 | -0.21 | -0.81, 0.39 | -0.36 | -0.73, 0.01 |
|  |  |  |  |  |  |  |  |  |

^a^ATE: Average treatment effect, i.e. the estimated average effect of weekly social contact within the sample after adjusting for education, partner status, wealth and all other confounders listed in Table 1. These estimates assume no residual confounding or reverse causation.
