# Supplementary material for: Social contact and inequalities in depressive symptoms and loneliness among older adults: A mediation analysis of the English Longitudinal Study of Ageing
Source: SSM Popul Health. 2021 Jan 12;13:100726. doi: 10.1016/j.ssmph.2021.100726 (PMC7820553; doi:10.1016/j.ssmph.2021.100726)
Supplement: Multimedia component 3 [file mmc3.docx]

**Supplementary Table 3: Continuous score estimates for effects of education, partner status and wealth on depressive symptoms and loneliness**

|  | **Low Education**  **(vs High Education)** | | | | **Lives Alone**  **(vs with Partner)** | | | | **Lowest Wealth Quintile**  **(vs Highest Quintile)** | | | |
| --- | --- | --- | --- | --- | --- | --- | --- | --- | --- | --- | --- | --- |
|  | *Age <65*  *N=1,578* | | *Age 65+*  *N=4,026* | | *Age <65*  *N=1,578* | | *Age 65+*  *N=4,026* | | *Age <65*  *N=1,578* | | *Age 65+*  *N=4,026* | |
|  | ß | 95% CI | ß | 95% CI | ß | 95% CI | ß | 95% CI | ß | 95% CI | ß | 95% CI |
| *CES-D Depressive symptoms* |  |  |  |  |  |  |  |  |  |  |  |  |
| ATE estimate^a^ | 0.42 | 0.14, 0.69 | 0.32 | 0.18, 0.45 | 1.43 | 1.04, 1.81 | 0.66 | 0.49, 0.83 | 1.05 | 0.59, 1.51 | 0.82 | 0.27, 1.38 |
| CDE estimate^b^  (<weekly in-person contact) | 0.35 | -0.38, 1.09 | 0.21 | -0.20, 0.62 | 0.75 | -0.16, 1.66 | 0.55 | 0.05, 1.05 | 0.97 | -0.52, 2.46 | 0.66 | -0.38, 1.73 |
| CDE estimate^b^  (weekly remote contact) | -0.21 | -0.94, 0.53 | 0.21 | 0.05, 0.38 | 2.14 | 0.81, 3.48 | 0.58 | 0.28, 0.83 | 0.69 | -0.12, 1.50 | 0.78 | 0.21, 1.35 |
| *UCLA Loneliness* |  |  |  |  |  |  |  |  |  |  |  |  |
| ATE estimate^a^ | 0.30 | 0.09, 0.52 | 0.24 | 0.14, 0.34 | 1.44 | 1.16, 1.71 | 0.93 | 0.80, 1.06 | 0.95 | 0.19, 1.71 | 0.70 | 0.19, 1.21 |
| CDE estimate^b^  (<weekly in-person contact) | 0.07 | -0.49, 0.60 | 0.09 | -0.28, 0.47 | 1.05 | 0.37, 1.74 | 1.34 | 0.86, 1.82 | 0.03 | -0.81, 0.88 | 0.47 | -0.16, 1.09 |
| CDE estimate^b^  (weekly remote contact) | -0.08 | -0.62, 0.47 | 0.18 | 0.05, 0.31 | 1.81 | 0.90, 2.72 | 0.91 | 0.74, 1.07 | 0.51 | -0.44, 1.45 | 0.51 | 0.01, 1.01 |
|  |  |  |  |  |  |  |  |  |  |  |  |  |

^a^ATE: Average Treatment Effect, i.e. the estimated average effect of each exposure, after adjusted for pre-exposure confounders (see Table 1). These estimates (and the CDE estimates) assume no residual confounding or reverse causation.

^b^CDE: Controlled Direct Effect, i.e. an estimate of the effect of each exposure if social contact were set to the value shown in parentheses. CDE estimates allow for interactions between social contact and the exposure and additionally adjust for differences in post-exposure confounders that were not due to the exposure (see Table 1).
