# Supplementary material for: Social contact and inequalities in depressive symptoms and loneliness among older adults: A mediation analysis of the English Longitudinal Study of Ageing
Source: SSM Popul Health. 2021 Jan 12;13:100726. doi: 10.1016/j.ssmph.2021.100726 (PMC7820553; doi:10.1016/j.ssmph.2021.100726)
Supplement: Multimedia component 4 [file mmc4.docx]

**Supplementary Table 4: Estimates for effects of partner status on depressive symptoms and loneliness with socioeconomic variables positioned as pre-exposure confounders**

|  | **Lives Alone**  **(vs with Partner)** | | | |
| --- | --- | --- | --- | --- |
|  | *Age <65*  *N=1,578* | | *Age 65+*  *N=4,026* | |
|  | OR | 95% CI | OR | 95% CI |
| *CES-D Depressive symptoms* |  |  |  |  |
| ATE estimate^a^ | 1.96 | 1.31-2.94 | 1.68 | 1.40-2.07 |
| CDE estimate^b^  (<weekly in-person contact) | 0.86 | 0.30-2.48 | 1.31 | 0.70-2.44 |
| CDE estimate^b^  (weekly remote contact) | 3.01 | 1.72-5.27 | 1.51 | 1.13-2.00 |
| *Sometimes/Often Feels Lonely* |  |  |  |  |
| ATE estimate^a^ | 4.73 | 3.18-7.04 | 4.40 | 3.65-5.30 |
| CDE estimate^b^  (<weekly in-person contact) | 3.16 | 1.00-10.04 | 5.02 | 2.76-9.14 |
| CDE estimate^b^  (weekly remote contact) | 5.82 | 3.67-9.23 | 4.71 | 3.64-6.08 |
| *UCLA Loneliness* |  |  |  |  |
| ATE estimate^a^ | 3.72 | 2.38-5.80 | 3.44 | 2.79-4.24 |
| CDE estimate^b^  (<weekly in-person contact) | 3.26 | 0.93-11.45 | 6.08 | 3.27-11.30 |
| CDE estimate^b^  (weekly remote contact) | 4.37 | 2.55-7.49 | 3.71 | 2.76-4.99 |
|  |  |  |  |  |

^a^ATE: Average Treatment Effect, i.e. the estimated average effect of partner status, after adjusted for pre-exposure confounders (see Table 1) with socioeconomic confounders (wealth, housing tenure, economic activity, and social class) re-positioned as pre-exposure confounders. These estimates (and the CDE estimates) assume no residual confounding or reverse causation.

^b^CDE: Controlled Direct Effect, i.e. an estimate of the effect of partner status if social contact were set to the value shown in parentheses. CDE estimates allow for interactions between social contact and partner status and additionally adjust for differences in post-exposure confounders that were not due to partner status (see Table 1).
